# Accidental Detection of Persistent Left Superior Vena Cava During Central Venous Port Placement

**DOI:** 10.7759/cureus.49478

**Published:** 2023-11-27

**Authors:** Shigenori Masaki, Takashi Kawamoto

**Affiliations:** 1 Surgery and Gastroenterology, Miyanomori Memorial Hospital, Sapporo, JPN; 2 Neurosurgery, Miyanomori Memorial Hospital, Sapporo, JPN

**Keywords:** thoracic vein, anomaly, central venous port, central venous catheter, plsvc, persistent left superior vena cava

## Abstract

Persistent left superior vena cava (PLSVC) is a rare abnormality of the thoracic vein that is often detected incidentally during central venous catheter insertion. We present the case of an 85-year-old female with PLSVC that was accidentally detected during central venous port placement. The left subclavian vein was punctured using the supraclavicular approach. Intraoperative fluoroscopy showed that the guidewire had descended through the left chest, suggesting PLSVC. Intraoperative computed tomography and venography confirmed that the PLSVC drained into the coronary sinus. In this case, the PLSVC and right superior vena cava (RSVC) coexisted, with no bridging veins. The diameter of the PLSVC was extremely small compared to that of the RSVC; therefore, catheter placement in the PLSVC was avoided considering the risk of venous thromboembolism, and a catheter was placed in the RSVC. When clinicians encounter cases where the PLSVC and RSVC coexist during central venous catheter insertion, the diameter of the PLSVC should be considered when deciding whether to place the catheter in the PLSVC. If the diameter of the PLSVC is narrow, it may be safer to avoid catheter placement in the PLSVC and instead place the catheter in the RSVC, considering the risk of venous thromboembolism after catheterization.

## Introduction

Persistent left superior vena cava (PLSVC) is a rare anomaly of the thoracic vein, with a prevalence of 0.3-0.5% in the general population and 2-10% in patients with congenital heart disease [1, 2]. During the embryonic period, the primitive venous system comprises paired vitelline veins, umbilical veins, and cardinal veins. The caudal portion of the right superior cardinal vein, along with the right common cardinal vein, participate in the formation of the right superior vena cava (RSVC). Typically, the left common cardinal vein and the caudal portion of the left superior cardinal vein regress, transforming into the ligament of Marshall; however, failure of regression results in a PLSVC [3]. One of the hypotheses regarding the development of PLSVC suggests that the presence of anomalies, such as an atrioventricular septal defect, leads to a reduction in left atrial pressure and inadequate left atrium development, contributing to the formation of PLSVC [1-3].

In most cases, the PLSVC connects to the coronary sinus (CS) and drains into the right atrium, whereas, in approximately 8-10% of cases, it drains into the left atrium or pulmonary veins, which may involve a right-to-left shunt [1-4]. It contributes to approximately 20% of the total venous return from the left side of the head, neck, and arm [3] and in 80-90% of PLSVC cases, the RSVC coexists with the PLSVC [2, 4]; among them, 65% have no communicating branch between the two [3].

PLSVC is often asymptomatic and detected incidentally during central venous (CV) catheter or pacemaker placement. Cases of CV catheter placement in PLSVC have been reported [5]; however, the safety of long-term CV catheter placement in the PLSVC has not yet been sufficiently established. When a clinician encounters a case in which both the PLSVC and RSVC coexist during CV catheter placement, a decision on whether to place the CV catheter in the PLSVC or RSVC is required. Herein, we report a case of the coexistence of the PLSVC and RSVC, which was incidentally encountered during CV port placement.

## Case presentation

**Figure 1:**
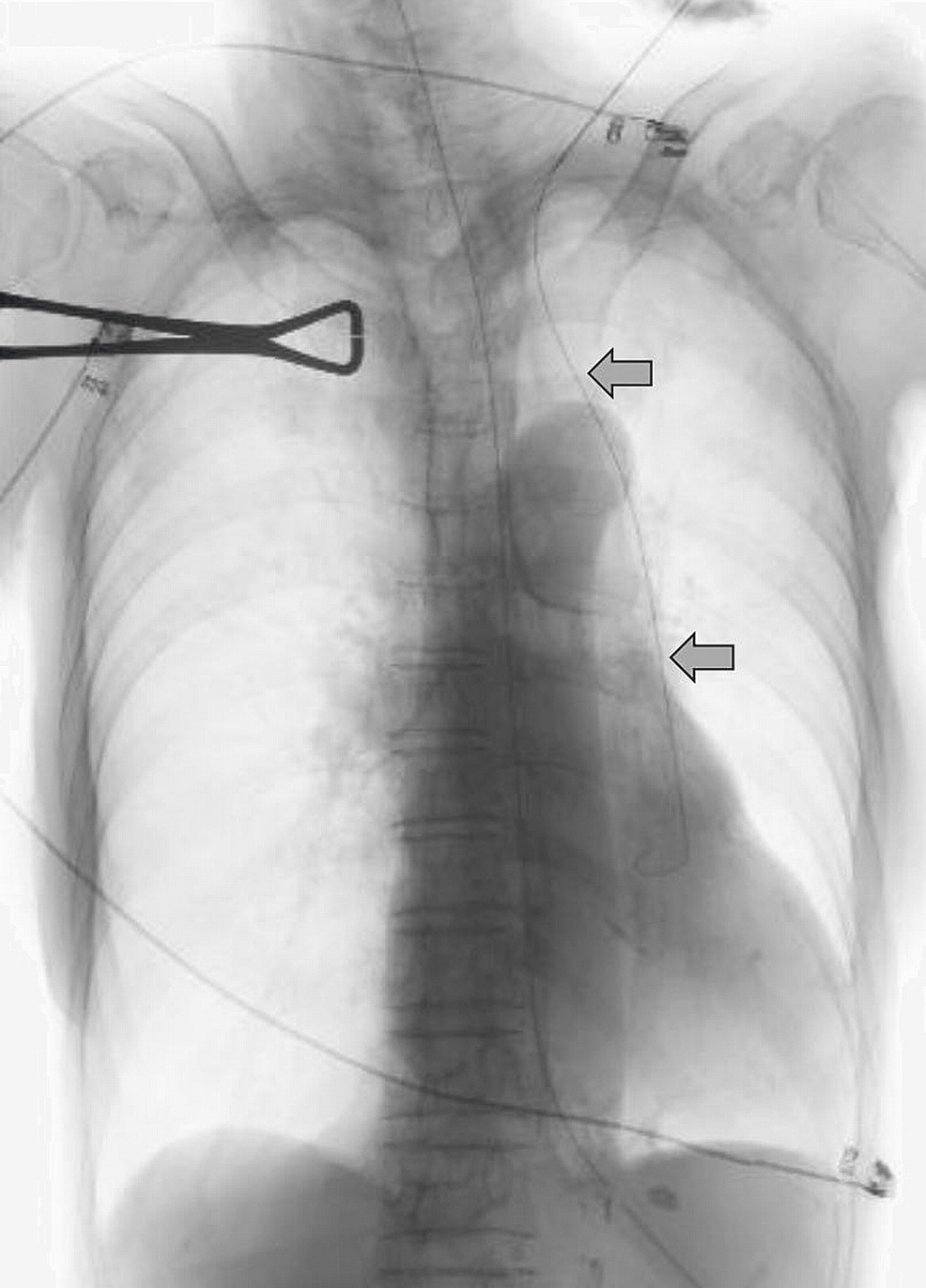
Intraoperative fluoroscopy findings. The guidewire (arrow) descended into the left side of the chest.

An 85-year-old female with dysphagia resulting from the after-effects of cerebral infarction received nasogastric tube feeding; however, gastroesophageal reflux and recurrent aspiration pneumonia made it difficult to continue nasogastric tube feeding. The patient was referred to our hospital for a CV port placement for parenteral nutrition. The patient had no history of congenital heart disease. The left subclavian vein was punctured using a supraclavicular approach under ultrasonographic guidance. Intraoperative fluoroscopy showed that the guidewire had advanced caudally along the left margin of the descending aorta, suggesting PLSVC (Figure 1).

**Figure 2:**
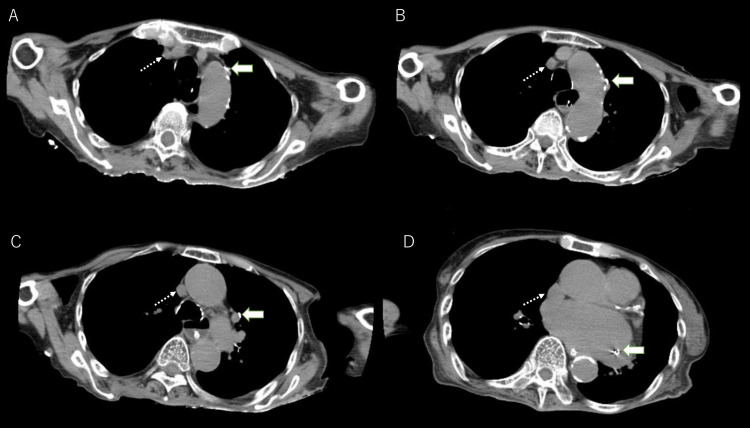
Intraoperative CT images with the guidewire inserted into the PLSVC. (A) PLSVC (solid arrow) and right superior vena cava (RSVC, dotted arrow) coexited. (B) PLSVC was extremely thin compared to RSVC. (C) There were no bridging veins between PLSVC and RSVC. (D) The PLSVC entered the CS.

Intraoperative computed tomography (CT) revealed the presence of PLSVC (Figure 2A-2D).

**Figure 3:**
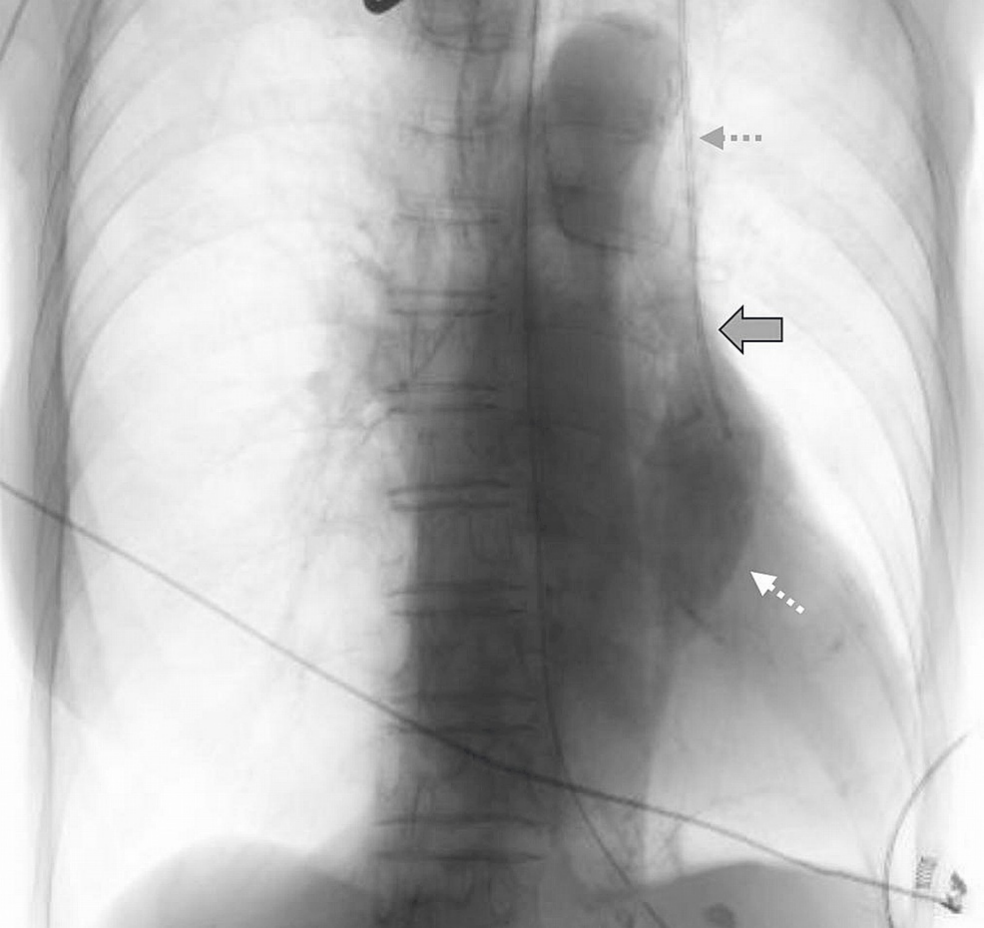
Venography findings. The catheter (outer diameter 1.5 mm, gray dotted arrow) was inserted into the PLSVC for venography. The PLSVC (solid arrow) drained into the right atrium through the dilated CS (white dotted arrow).

The diameter of the PLSVC measured on CT was small, with a minimum diameter of 3.4 mm. A thicker RSVC was observed on CT, and no communication branch between the PLSVC and RSVC was identified. Venography revealed that the PLSVC had drained into the dilated CS (Figure 3).

**Figure 4:**
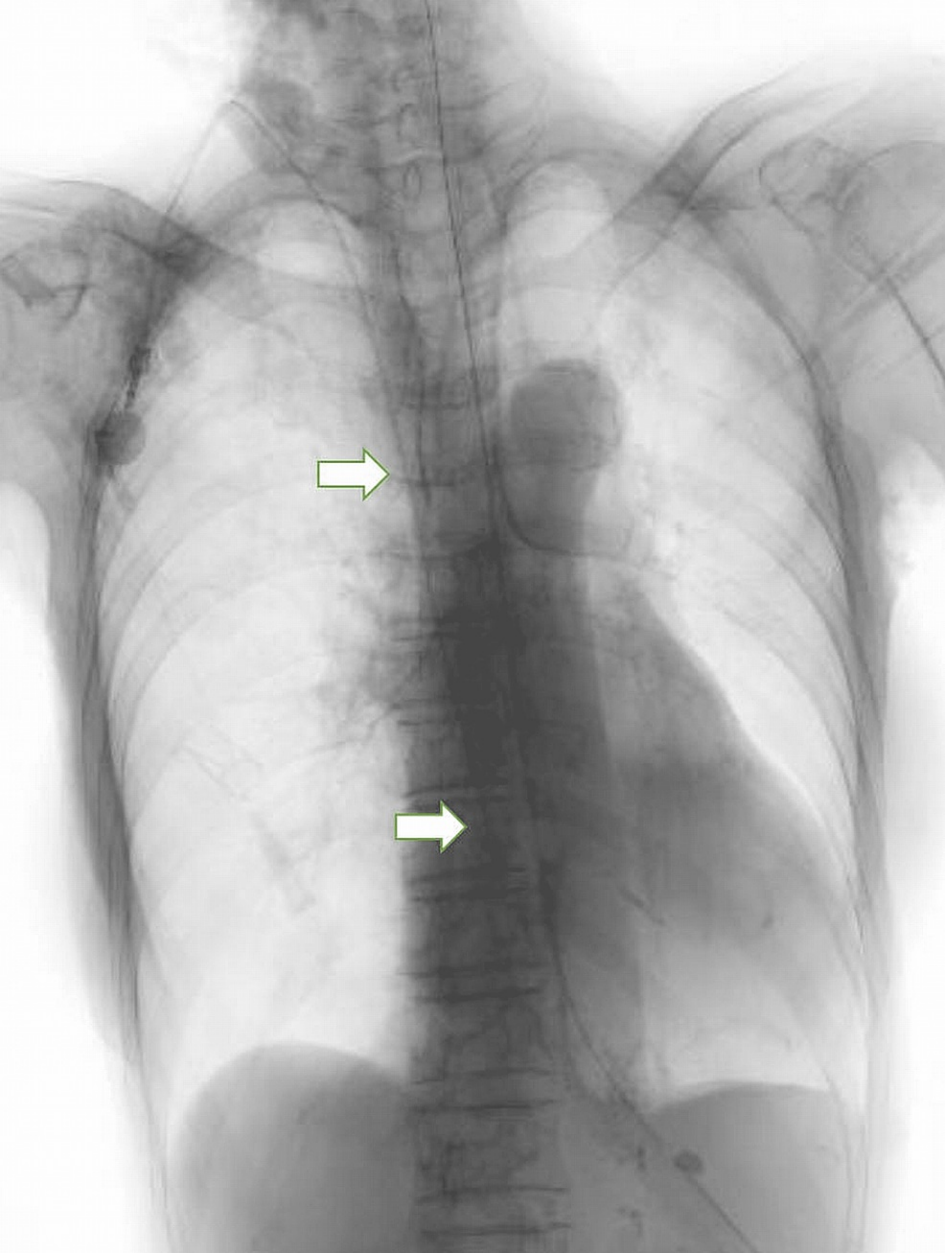
Fluoroscopy images after central venous (CV) port placement. The CV port catheter was placed in the RSVC (arrow).

The outer diameter of the CV port catheter that we planned to place was 2.5 mm, and the ratio of the catheter diameter to the PLSVC diameter was 74%. Therefore, the patient's PLSVC was considered unsuitable for catheter placement due to its narrow size, which posed a risk of catheter-related venous thromboembolism. We decided to avoid placing the catheter in the PLSVC and instead chose to place it in the RSVC. Finally, a catheter was placed in the RSVC through the right internal jugular vein puncture (Figure 4).

The patient was discharged from our hospital on postoperative day 11 without complications.

## Discussion

In this report, we describe a case of PLSVC incidentally detected during CV port placement. Owing to the thinness of the PLSVC, catheter placement into the PLSVC was avoided, and a catheter was ultimately placed into the RSVC.

Variations of the superior vena cava are categorized into four types based on the CV catheter position on chest radiograph: type I represents normal anatomy; type II indicates only PLSVC; type IIIa signifies both the RSVC and PLSVC with connection; and type IIIb denotes the RSVC and PLSVC without connection [6, 7]. Our case corresponded to type IIIb in this classification. In cases where the RSVC and PLSVC coexist, such as in type IIIa and type IIIb cases, the choice between the RSVC and PLSVC for long-term CV catheter placement remains controversial.

Over the past 20 years, a total of 21 CV port catheter placements in the PLSVC have been reported [8-12]; for 20 cases, this placement was chosen for chemotherapy, while it was chosen for parenteral nutrition in one case. The longest catheter indwelling period was 756 days, with no catheter-related problems reported during that period [9]. In one of the 21 cases, recurrent cerebral embolism associated with PLSVC drainage into the left atrium was observed [12]. PLSVC catheterization has been reported to be associated with arrhythmia, venous stenosis, thrombosis, cardiac tamponade, and cardiac arrest [10, 13]. Therefore, the feasibility of long-term catheterization in PLSVC should be carefully determined [9, 10, 12, 13].

When considering long-term CV catheter placement, it is important to consider the ratio of the catheter diameter to vein diameter as the catheter can impede blood flow, thereby contributing to thrombosis [14]. The obstruction of blood flow depends on the size of the catheter and the vein, and to reduce the risk of catheter-related venous thromboembolism, it is desirable to maintain a catheter-to-vein diameter ratio of 45% or less [11, 14]. In this case, the ratio of the catheter to PLSVC diameter was 74%; therefore, a decision was made to avoid catheter placement in the PLSVC. When a clinician encounters a case of PLSVC during the placement of a CV catheter, it is important to consider the ratio of the catheter diameter to PLSVC diameter to determine whether catheter placement in the PLSVC is permissible.

## Conclusions

PLSVC is a rare anomaly of the thoracic vein that clinicians often encounter incidentally during the insertion of a CV catheter. The feasibility of placing a CV catheter in the PLSVC should be determined by considering the diameter of the catheter and PLSVC. If the PLSVC is too narrow and a thicker RSVC is present, catheter placement in the PLSVC should be avoided due to the risk of venous thromboembolism; placement in the RSVC should instead be considered.

## References

[1] Batouty NM, Sobh DM, Gadelhak B, Sobh HM, Mahmoud W, Tawfik AM (2020). Left superior vena cava: cross-sectional imaging overview. Radiol Med.

[2] Savu C, Petreanu C, Melinte A (2020). Persistent left superior vena cava - accidental finding. In Vivo.

[3] Azizova A, Onder O, Arslan S, Ardali S, Hazirolan T (2020). Persistent left superior vena cava: clinical importance and differential diagnoses. Insights Imaging.

[4] Sabzwari SR, Kimber J, Godil SA, Khan W, Mir J (2020). Pacemaker implantation in patient with persistent left superior vena cava and absent right superior vena cava. Cureus.

[5] Lopes Morgado F, Saraiva B, Blanco Torres C, Correia J (2021). Persistent left superior vena cava: a finding after central venous catheterization. Eur J Case Rep Intern Med.

[6] Schummer W, Schummer C, Fröber R (2003). Persistent left superior vena cava and central venous catheter position: clinical impact illustrated by four cases. Surg Radiol Anat.

[7] Evers C, Gazis A, Thuss-Patiance W, Kretzschmar A (2017). Misplacement of a port catheter: a differentiated view. Case Rep Radiol.

[8] El-Helou E, Zaiter M, Shall A, Sleiman Y, Liberale G, Pop CF (2022). Persistent left superior vena cava associated with right aberrant subclavian artery detected during totally implantable vascular access device insertion. Surg J (N Y).

[9] Jheengut Y, Fan B (2021). Intraoperative identification of persistent left superior vena cava with intracavitary electrocardiogram during venous port insertion: a report of eight cases. J Vasc Access.

[10] Zhou Q, Murthy S, Pattison A, Werder G (2016). Central venous access through a persistent left superior vena cava: a case series. J Vasc Access.

[11] Zhou RN, Ma XB, Wang L, Kang HF (2022). Accidental venous port placement via the persistent left superior vena cava: two case reports. World J Clin Cases.

[12] Dinasarapu CR, Adiga GU, Malik S (2010). Recurrent cerebral embolism associated with indwelling catheter in the presence of anomalous neck venous structures. Am J Med Sci.

[13] Puspitasari M, Sinorita H, Bagaswoto HP, Kuswadi I, Prasanto H, Wardhani Y, Kurniawan WT (2020). Persistent left superior vena cava identified after hemodialysis catheter insertion: a case report. Int Med Case Rep J.

[14] Sharp R, Carr P, Childs J (2021). Catheter to vein ratio and risk of peripherally inserted central catheter (PICC)-associated thrombosis according to diagnostic group: a retrospective cohort study. BMJ Open.

